# Periodic fever, aphthous stomatitis, pharyngitis, adenitis: clinical manifestations, response to steroids, and a practice of online diagnosis and follow-up

**DOI:** 10.3389/fimmu.2026.1962224

**Published:** 2026-09-24

**Authors:** Zhishan Li, Keyou Wen, Cong Liang, Yu Li, Zhiyong Ke

**Affiliations:** 1 Department of Pediatrics, The First Affiliated Hospital of Sun Yat-sen University, Guangzhou, Guangdong, China; 2 Zhongshan School of Medicine, Sun Yat-sen University, Guangzhou, Guangdong, China

**Keywords:** internet, manifestation, online, periodic fever, PFAPA, response to steroids

## Abstract

**Background:**

Periodic fever, aphthous stomatitis, pharyngitis, and adenitis (PFAPA) syndrome, although not rare, is frequently underdiagnosed. This might be related to the fact that some primary care physicians are not familiar with this syndrome. With the application of network technology, internet tools may also be used for remote diagnosis and follow-up of PFAPA. Herein, we present our experience regarding online diagnosis and management, and provide a more detailed clinical profile of PFAPA in children.

**Methods:**

All children with long-standing recurrent fever, including those seen at our hospital (clinic group) and those managed through the Haodf.com online consultation platform (online group), were enrolled and followed up. Children diagnosed with PFAPA were retrospectively analyzed, with particular attention to response to corticosteroid therapy during episodes. Clinical manifestations were compared between the two groups.

**Results:**

Among 1,211 patients with PFAPA, febrile episodes lasting 4.0 days (3.0, 4.5) recurred at intervals of 30.0 days (20.0, 30.0), accompanied by exudative tonsillitis (85.5%), leukocytosis (88.6%) and elevated C-reactive protein (95.7%). Prednisone aborted episodes within 3.0 hours (1.0, 4.0); however, 54.2% of patients experienced shortened intervals after prednisone treatment, 38.2% showed no change in intervals, and 7.6% had prolonged cycles. Recrudescence within 24~72 hours was observed in 29.0% of patients who received a single low dose of prednisone. Clinical manifestations were generally similar between the clinic and online groups. At the time of diagnosis, children had experienced recurrent episodes for a median of 12.0 months (6.0, 12.0), yet only 18.4% had previously been suspected of or diagnosed with periodic fever or PFAPA.

**Conclusions:**

Oral prednisone is highly effective in aborting attacks but may shorten the interval between episodes in most patients. Underdiagnosis remains common. Online diagnosis and follow-up represent feasible and convenient approaches to PFAPA management.

## Introduction

Febrile illnesses are the most common conditions in childhood, and some children experience recurrent fevers with such regularity that they are termed periodic fevers. Periodic fever encompasses a broad category of autoinflammatory diseases, including classic hereditary periodic fevers such as familial Mediterranean fever, hyperimmunoglobulin D syndrome, and Muckle-Wells syndrome, as well as PFAPA (1–3). In recent years, with the widespread use of genetic testing, the concept of monogenic inherited autoinflammatory diseases has emerged, and the spectrum of these conditions continues to expand (2–6). Unlike classic hereditary periodic fevers, which are more prevalent in specific populations, PFAPA is believed to occur without racial predilection. Since its initial description in 1987 (7), PFAPA has gradually gained recognition, with an increasing number of reports in the literature (8–14). Precise data on the incidence of PFAPA remain limited. Reports from north Europe showed the annual incidence of 2-2.6/10000 in children <5 years (11, 15, 16). These extremely low incidences do not match the impression we have in our daily clinical practice. The true rate may be higher. A controlled study from the United States investigated family history and inheritance patterns in this syndrome; among siblings and parents in the control group (healthy control subjects), 2% and 1%, respectively, reported a history of recurrent fevers meeting diagnostic criteria for PFAPA (17). These incidence rates may serve as a reference for the general population, suggesting that this syndrome is not rare in essence. However, in our clinical practice, we have found that underdiagnosis is remarkably common. This high rate of underdiagnosis may be attributable to insufficient awareness among clinicians. In recent years, the rapid advancement of internet technology has enabled remote diagnosis and follow-up in patients with long-lasting diseases. Our research group has evaluated and followed a large cohort of children with periodic fever, most of whom were from online medical platforms. Here we retrospectively summarize the clinical manifestations of children with PFAPA, with particular emphasis on the response to corticosteroid therapy during acute episodes. We also compared the clinical manifestations of PFAPA children evaluated in the outpatient clinic with those diagnosed and followed up online, with the aim of assessing the reliability of online management.

## Methods

### Patients enrollment and follow-up

Children under 18 years of age with regular recurrent fever were enrolled for follow-up. Patients presenting with overt symptoms of respiratory or gastrointestinal infection such as rhinorrhea, cough, or diarrhea were excluded. The definition of fever regularity was based on the CARRA-PFAPA criteria (18). The date of each previous febrile episode and the accompanying symptoms, including pharyngitis, tonsillar exudation, oral ulcers, cervical lymphadenopathy, chest pain, abdominal pain, arthralgia, and rash, were systematically documented. Family history was traced among parents, siblings, grandparents, uncles and aunts, with particular emphasis on whether these relatives had experienced recurrent fever or frequent oral ulcers from childhood through adulthood. Laboratory findings were also comprehensively recorded, including complete blood count, urinalysis, C-reactive protein (CRP), procalcitonin, antistreptolysin, EBV-DNA, immunoglobulin levels, lymphocyte subsets, and genetic testing results. The majority of patients in the clinic group additionally underwent cytokine testing when they attended during febrile episodes.

Enrolled patients were divided into two groups: the clinic group (in-person visits) and the online group (telemedicine consultations). Patients in the clinic group were followed up via WeChat after their initial visit, with intermittent in-person re-evaluations. Patients in the online group were followed up through the Haodf.com platform, whose physical examinations and laboratory tests were performed at local medical institutions according to standardized guidance, and results were reported by guardians online. The attending physician then provided management recommendations based on the actual conditions and resources available locally. Online patients continued to be followed up on the Haodf.com platform. During each febrile episode, guardians were instructed to report online and manage the episode according to standardized guidance. When necessary, physical examinations and auxiliary laboratory tests were conducted by local medical institutions.

### Diagnosis

The diagnostic criteria for PFAPA were established by the Childhood Arthritis and Rheumatology Research Alliance (CARRA) PFAPA Work Group (Consensus Treatment Plans) (18). Periodic neutropenia could be easily ruled out through blood routine tests. For children who met the definition of periodic fever at enrollment, it was recommended that they take a single oral dose of prednisone during the next febrile episode, refraining from other medications, particularly antibiotics, except for antipyretics. The response to a single dose of prednisone (2mg/kg/dose, not more than 60mg) was then observed; patients whose fever resolved without recurrence were considered to meet the diagnostic criteria. Since some patients reported that attacks could be eliminated even with a very small dose of prednisone on their own before diagnosis of PFAPA, in the later phase of the study, a lower dose of prednisone (1 mg/kg per dose) was attempted. For patients whose fever recurred more than 24 hours after initial resolution, an additional dose of prednisone was administered; if the fever resolved again and did not recur, these patients were also considered to meet the criteria. All children diagnosed with periodic fever were followed up for six months to carefully exclude other febrile disorders. Some patients underwent whole-exome sequencing to rule out hereditary periodic fever or monogenic autoinflammatory diseases. Patients with variants classified as benign by bioinformatics analysis were deemed consistent with PFAPA. Patients with variants of uncertain significance or pathogenicity were also considered not inconsistent with PFAPA if they met the following conditions: heterozygous carriers of autosomal recessive (AR) variants, female heterozygous carriers of X-linked recessive (XLR) variants, or patients with autosomal dominant (AD) variants whose carrier parents were asymptomatic. All other cases with suspected genetic abnormalities were excluded from the case analysis in this article.

### Data collection and statistical analysis

The febrile cycle was defined as the interval from the first day of fever onset to the first day of the subsequent febrile episode. As the cycle was not entirely constant and occasional episodes might not occur as expected, the relatively stable interval between episodes was adopted as the cycle length. As we observed that a considerable proportion of children experienced shortened cycles after corticosteroid administration, the cycle lengths reported were all measured prior to steroid use. The duration of each episode was defined by the number of days with sustained fever. For some children who received steroids at the onset of fever due to previous history of febrile seizures, or those with undocumented medication histories at local clinics suggesting possible steroid use, their episode durations were excluded from analysis. Accompanying symptoms during episodes were recorded at enrollment. Symptoms that had not appeared prior to enrollment but newly emerged during subsequent episodes in the follow-up period were also retrospectively added to the symptom profile. Older children could accurately report arthralgia or abdominal pain, whereas younger children could not; therefore, these symptoms were not recorded for children under 2 years of age. This study was primarily retrospective in design, and some patients had missing data due to inability to obtain accurate records. Statistical analysis was performed using SPSS Statistics 25. Measurement data are expressed as median (interquartile range), as the data did not follow a normal distribution. The chi-square test and the Mann-Whitney test were used for comparisons between groups and tests for the associations between factors.

## Results

### Diagnostic profile

From February 1, 2021 to December 31, 2025, a total of 1,639 children with regular periodic fever were enrolled. Of these, 270 were lost to follow-up and excluded. Among the remaining 1,369 patients under follow-up, 2 confirmed to have factitious fever, 3 had cyclic neutropenia, 1 had polycystic kidney disease and recurrent urinary tract infections, and 3 had deep oral ulcers suspicious for Behçet’s disease (2 of whom also had genital ulcers). One patient with multiple colonic ulcers on endoscopy was considered to have inflammatory bowel disease; one with refractory multiserositis was considered to have an unclassified autoinflammatory disease; two were considered to have food allergy; and one had coincidentally regular respiratory tract infections (**Figure 1**). Among the remaining 1,355 patients, 29 lacked height data and 80 had height exceeding 2 standard deviations for age and gender, and were therefore excluded. Some patients underwent whole-exome sequencing; 257 received reports, of whom 35 had questionable findings and were excluded.

**Figure 1:**
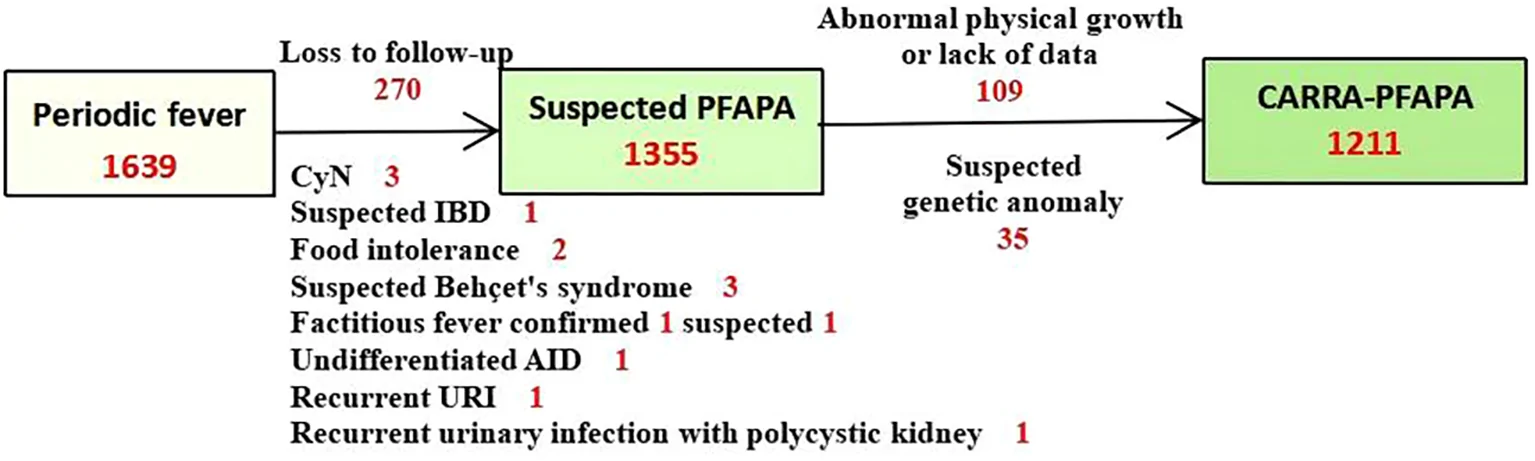
Flowchart of patient enrollment and exclusion.

A total of 1,211 patients were included in the final analysis, with a male predominance (723, 59.7%). At the time of enrollment and initiation of follow-up, these children had already experienced recurrent febrile episodes for a median of 12.0 (6.0, 12.0) months, ranging from 1 month to 178 months. Only 223 patients (18.4%) had previously been suspected of or diagnosed with periodic fever or PFAPA at other medical institutions.

### Clinical manifestations

The majority of patients (926, 73.4%) experienced onset of recurrent fever before 5 years of age; only 23 cases had onset at 10 years or older (**Figure 2**). The minimum age at onset was 1 month, and the maximum was 174 months. The febrile cycle was 30.0 (20.0, 30.0) days, ranging from 6 to 90 days; 9 patients had frequent episodes with cycles shorter than 10 days. Each episode lasted a median of 4.0 (3.0, 4.5) days, ranging from 2 to 9 days. During history-taking and follow-up, 125 patients (10.3%) experienced remission periods lasting from several months to several years, with a median duration of 12 (6, 24) months (range: 3 to 71 months), after which recurrent episodes resumed.

**Figure 2:**
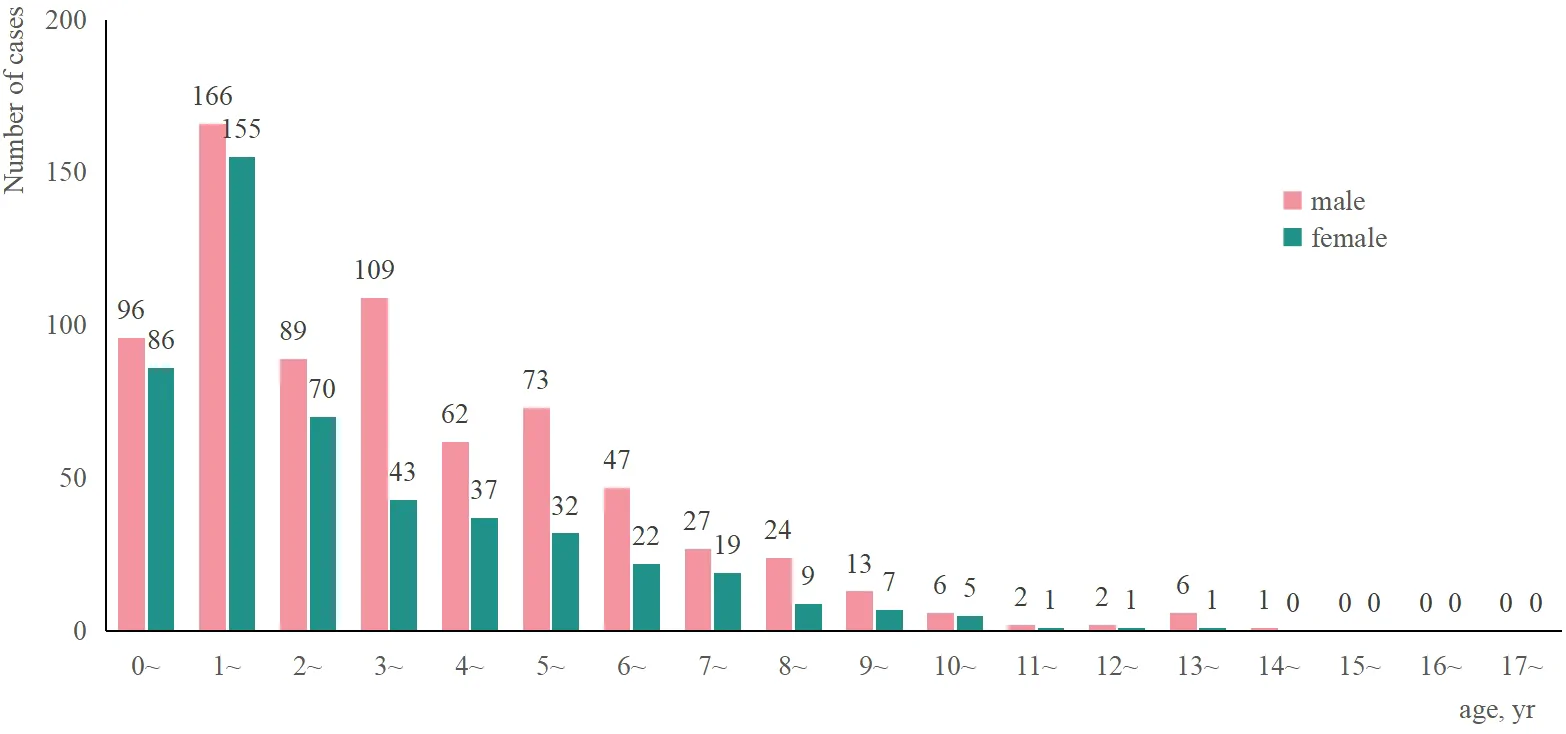
Age at onset and gender distribution of patients.

In addition to fever during episodes, the vast majority of patients presented with tonsillar exudation; nearly half had oral ulcers, cervical lymphadenopathy, and abdominal pain, while a minority experienced limb pain or arthralgia (**Table 1**). The majority of patients had Leukocytosis and elevated CRP levels during episodes. These symptoms and laboratory abnormalities returned to normal after resolution of the episode. Among the 84 patients in in-person group who underwent cytokine testing during febrile episodes, IL-2 was elevated in 0%, IL-4 in 7.1%, IL-6 in 96.4%, IL-10 in 48.8%, IL-17 in 3.6%, interferon-γ in 60.7%, and TNF in 2.4%. Some patients underwent repeat testing during the inter-episode period, and all results normalized.

**Table 1:** Clinical features of the patients.

Characteristics	Total	Clinic group	Online group
Gender (male/female)	723/488	132/86	591/402
Age of onset, months	30.0 (13.8-59.0)	36.0 (18.0-62.0)	28.0 (13.0-58.0)*
Cycle length, days	30.0 (20.0-30.0)	30.0 (20.0-30.0)	30.0 (20.0-30.0)
Duration of attack, days	4.0 (3.0-4.5)	4.0 (3.0-4.5)	4.0 (3.0-4.5)
Leukocytosis, %	88.6% (1157)	90.0% (210)	88.3% (946)
Elevated CRP, %	95.7% (1152)	94.7% (209)	96.0% (943)
Exudative tonsillitis, %	85.5% (1182)	92.6% (215)	84.0% (967)*
Aphthous ulcer, %	50.0% (1187)	57.3% (218)	48.4% (969)*
cervical lymphadenitis, %	42.7% (1158)	50.5% (214)	40.9% (944)*
Abdominal pain, %	41.3% (995)	34.6% (205)	43.0% (790)*
Arthralgia or limb pain, %	23.4% (986)	23.7% (207)	23.4% (779)
Positive family story^#^, %	38.3% (1190)	33.6% (217)	39.4% (973)

Measurement data were expressed as the median (interquartile range). The number of evaluable cases was indicated in parentheses in each item of count data.

*P<0.05

^#^Histories of recurrent fever without symptoms of respiratory or gastrointestinal infections, including but not limited to PFAPA.

### Response to prednisone

At onset of suspected episodes (axillary temperature exceeding 38 °C without respiratory symptoms such as rhinorrhea or cough, or gastrointestinal symptoms such as diarrhea; or fever accompanied by tonsillar exudation), patients were instructed to take a single oral dose of prednisone. In 131 patients who were specifically monitored for cycle changes after steroid administration without preventive measures, 71 (54.2%) experienced shortened cycles, 50 (38.2%) had unchanged cycles, and 10 (7.6%) had prolonged cycles. Some patients were observed for antipyretic response to oral prednisone during episodes; fever resolved within 1–4 hours after administration (median: 3.0 hours). From August 2025 onward, we recommended a single lower dose of prednisone (approximately 1 mg/kg per dose) during episodes. Among 124 patients with careful records, 88 did not experience recurrence, while 36 (29.0%) had recrudescence. Fever recurrence typically occurred between 24 and 72 hours after initial resolution, with a median of 42 hours (30.0, 63.0). In the follow-up period 12 patients had fever resolution lasting less than 12 hours after oral prednisone, and were subsequently confirmed to have influenza or respiratory/gastrointestinal viral infections.

### Family history

When the presenting complaint was recurrent fever without respiratory or gastrointestinal infection symptoms, 456 cases (38.3%) among the 1,190 children whose family history was reliably obtained had a positive family history. A total of 389 children were recorded with a history of long-term and recurrent fever in their parents (191 cases only in the father, 180 cases only in the mother, and 18 cases in both parents). A positive history in siblings was found in 30 cases, of which 20 were positive in siblings alone and 10 were positive in both siblings and parents. A positive history in grandparents was found in 26 cases, of which 17 were positive in grandparents alone and 9 were positive in both grandparents and parents on the same lineage. A positive history in uncles/aunts was found in 11 cases, of which 6 were positive in uncles/aunts alone and 5 were positive in both uncles/aunts and parents on the same lineage.

### Comparison of clinical manifestations between groups and correlation analysis

Most of the 218 in-person patients were from Guangdong Province (198, 90.8%), especially from Guangzhou City (84, 38.5%). The online patients came from 30 provincial administrative regions across China, with the highest proportion from Jiangsu, Zhejiang and Shanghai (258, 26.0%), while only (92, 9.3%) were from Guangdong Province. Both groups had a male predominance, accounting for 60.5% of the clinic group and 59.5% of the online group respectively. Patients in the clinic group had older age at onset compared with those in the online group. Overall, manifestations during episodes were similar, but objective symptoms such as tonsillar exudation, oral ulcers, and cervical lymphadenopathy were slightly more frequent in the clinic group, whereas reported abdominal pain was less common (**Table 1**).

The cohort was predominantly male; 723 male patients (81.7%) were followed up online, as were 488 female patients (82.4%). Clinical manifestations during episodes were similar between boys and girls, but boys appeared to have older age at onset (**Figure 2**) and were more likely to present with cervical lymphadenopathy during episodes.

Age at onset was positively correlated with local manifestations such as tonsillar exudation, oral ulcers, lymphadenopathy, and arthralgia, as well as with leukocytosis and elevated CRP during episodes; older age at onset was associated with more typical presentations. Age at onset was significantly negatively correlated with cycle length, but not with episode duration. No correlation was found with abdominal pain during episodes or family history.

The duration of disease at the time of enrollment and follow-up was positively correlated with tonsillar exudation and oral ulcers; the longer the disease course, the greater the likelihood of documenting these manifestations. It was also positively correlated with cycle length; longer disease duration was associated with progressively longer cycles. No correlation was found with episode duration, abdominal pain, arthralgia, lymphadenopathy, leukocytosis, or elevated CRP.

Leukocytosis during episodes was highly positively correlated with elevated CRP and positively correlated with tonsillar exudation, but negatively correlated with oral ulcers. No correlation was found with family history, lymphadenopathy, abdominal pain, or arthralgia.

## Discussion

PFAPA is not a rare disease, as mentioned before, but it is often overlooked in clinical practice. In this cohort, only a small proportion of children had been suspected of or diagnosed with periodic fever prior to enrollment in our follow-up program, and most children had experienced a prolonged disease course before final diagnosis. This syndrome is frequently overlooked or misdiagnosed in clinical settings across China, as most frontline physicians are not familiar with this condition. Some physicians confuse PFAPA with classic hereditary periodic fever syndromes. Since hereditary periodic fever is extremely rare in East Asian populations, local physicians lack vigilance toward periodic fever in general, which consequently extends to inadequate recognition of the relatively more common PFAPA. This lack of alertness leads to the common clinical practice of managing each episode in isolation rather than integrating multiple episodes into a unified diagnostic framework. Enhancing awareness of this disease will reduce the rate of missed diagnoses (9, 19, 20). Although PFAPA is a benign condition, its recurrent episodes continue to impose burdens on children, families, and national healthcare systems (12, 21–24). Quality of life for the child and family may will significantly improve after tonsillectomy or even simply upon confirmation of the PFAPA diagnosis (21, 22). For children with recurrent regular fever, particularly those accompanied by tonsillar exudation, evaluating multiple febrile episodes as an integrated whole and promptly recognizing PFAPA is undoubtedly essential.

As the name implies, PFAPA is characterized by periodic fever, aphthous stomatitis, pharyngitis, and cervical adenitis. The periodicity of fever is undoubtedly the most fundamental characteristic of PFAPA. In this cohort, the febrile cycle was approximately 30 days with episodes lasting 4 days, consistent with other studies (8, 11–14, 25–31). We have observed that the cycle in children with PFAPA was not constant; as the disease progressed, the cycle often exhibited a pattern of gradual shortening, stabilization, and subsequent gradual lengthening. We also found that a minority of patients experienced remission periods lasting from several months to several years during the prolonged disease course, similar to findings reported by other authors (14, 20).

In addition to regular periodic fever, pharyngitis, particularly tonsillar exudation, was the most common symptom. The vast majority of patients had leukocytosis and elevated CRP levels during an attack, and patients with tonsillar exudation during episodes more commonly had concomitant leukocytosis and elevated CRP. All these present the classic image of “purulent tonsillitis”. This has led to the prevailing misuse of antibiotics during PFAPA attacks before diagnosis. However, unlike true suppurative tonsillitis, a single oral dose of prednisone during a PFAPA episode generally terminated the entire episode, without the need for antibiotics. Besides the pharyngitis or tonsillitis, nearly half of the patients experienced oral ulcers, cervical lymphadenopathy, and abdominal pain during episodes, while a minority presented with musculoskeletal symptoms. These manifestations may vary in different regions or populations (32).

The response to corticosteroids is a key element in the diagnosis of PFAPA and even a clue to distinguish it from infection or other types of periodic fever (2, 26, 30, 33–35). While steroids effectively terminate PFAPA episodes, their antipyretic effect is not as rapid as that of non-steroidal antipyretics. After oral prednisone administration, fever typically begins to subside after 1–2 hours, with complete normalization of body temperature within 4–6 hours. It has been reported that lower doses of prednisone can resolve a single episode in the majority of children, although a minority experience recrudescence (8, 13, 14, 36, 37). We also observed that a small number of children who received a relatively low dose of prednisone experienced a recurrence of fever after the initial fever subsided. The timing of fever recrudescence is rather distinctive, typically occurring 24–72 hours after initial resolution, which does not prevent differentiation between a periodic fever episode and an infectious fever. Steroid administration during episodes does indeed alter the subsequent cycle (1, 3, 8, 13, 26). Most of the patients in this cohort experienced more frequent subsequent attacks when steroid were used to undo the episodes.

The pathogenesis of PFAPA remains incompletely understood. Familial clustering of the disease suggests that genetic factors play a role in the pathogenesis, while the cessation of recurrent attacks after tonsillectomy indicates that the local microenvironment of the tonsils is also involved in triggering the onset of PFAPA (8, 13, 21, 30). The most detailed description of family history comes from Manthiram’s research (17). However, as the history of PFAPA’s proposal is not long and the disease heals with age, it is clearly very difficult to identify whether the repeated fevers in childhood of today’s adults are PFAPA. Moreover, PFAPA is not yet widely recognized in China. Therefore, we defined a positive family history as recurrent fever in childhood without respiratory or gastrointestinal infection symptoms. In our study, more than one-third of patients had a positive family history of recurrent fever in their parents traceable to childhood, consistent with the report by Manthiram (17). It should be noted that relatives who have had repeated fevers during childhood cannot be understood as having had PFAPA before. The relatively low frequency of positive sibling histories may be attributable to the one-child policy, which limits the availability of siblings for family history assessment. Genetic testing is an important means of diagnosing autoinflammatory diseases, especially for hereditary periodic fever and monogenic autoinflammatory diseases (3, 4, 38). A variety of gene mutations related to autoinflammation have also been detected in children with PFAPA, but their role in the pathogenesis is controversial (3, 10, 27, 28, 39–42). Given the high cost of testing and the uncertainty in interpreting the test reports, we suggest that genetic tests should only be performed for patients suspected of having hereditary periodic fever or other monogenic autoinflammatory diseases (33, 38).

As a chronic, recurrent disease, PFAPA requires frequent follow-up visits. Modern internet technology has been widely applied, and online medical consultation platforms together with WeChat have made telemedicine follow-up a reliable reality. The majority of in-person patients are from Guangdong Province in the local area, while the online patients are from all over the country. Using internet tools can serve more patients from afar. The proportion of patients in the online group in East China is relatively high. We believe that this does not indicate a higher incidence rate in that area, but rather that people in economically developed regions may be better at using Internet tools. Our comparison revealed good consistency between in-person and online consultations. Objective signs such as tonsillar exudation and cervical lymphadenitis were slightly more common in the clinic group, possibly because we placed greater emphasis on identifying these signs, whereas online patients’ local physicians had not yet recognized or prioritized examination and documentation of PFAPA-related signs. Internet technology can not only assist in the identification and diagnosis of children with periodic fever, but also enable remote management guidance during episodes and play a role in subsequent prevention and follow-up. As far as we know, this represents the first large-scale attempt at online diagnosis and follow-up of PFAPA patients.

## Conclusions

The classic manifestation of PFAPA is recurrent regular fever accompanied by tonsillar exudation, and it has a unique response to prednisone treatment during an attack. The advanced application of modern internet technology can be utilized for close follow-up and guidance, providing substantial assistance in the diagnosis and management of children with PFAPA.

## Data Availability

The raw data supporting the conclusions of this article will be made available by the authors, without undue reservation.
